# Prevalence of Cannabidiol (CBD) Use Among Patients Taking Medications with Known Drug–Drug Interactions: A Cross-Sectional Analysis

**DOI:** 10.3390/jcm14217776

**Published:** 2025-11-02

**Authors:** Hunter Geneau, Michael Kovasala, Grant Brown, Simeon Holmes, Olivia Hime, Michael McNally, Michael McFayden, Kori Brewer, G. Kirk Jones

**Affiliations:** Brody School of Medicine, East Carolina University, Greenville, NC 27834, USA; kovasalak23@students.ecu.edu (M.K.); browngran23@students.ecu.edu (G.B.); holmessimeo23@students.ecu.edu (S.H.); himeo23@students.ecu.edu (O.H.); mcnallym18@students.ecu.edu (M.M.); mcfaydenm23@students.ecu.edu (M.M.); brewerk@ecu.edu (K.B.); jonesgi23@ecu.edu (G.K.J.)

**Keywords:** cannabidiol (CBD), adverse drug events (ADEs), drug-drug interactions (DDIs), patient safety, prescription medications, emergency department, patient awareness, public health, observational study

## Abstract

**Introduction**: Cannabidiol (CBD) is widely available over the counter for presumed medical and recreational purposes. Despite its non-psychoactive nature, CBD exhibits intrinsic pharmacological activity that may lead to potential adverse drug events (ADEs) and drug–drug interactions (DDI) with common prescription medications through cytochrome P450 inhibition. Due to their largely unregulated nature and widespread advertising, consumers who use CBD products may not be aware of these potential negative drug interactions. The purpose of this study was to determine how frequently patients who use CBD products concurrently take prescription medication with known drug–drug interaction (DDI) potential, and to identify specific therapeutic classes most commonly involved. **Methods**: In this cross-sectional study, a survey was distributed to patients and family members in the adult and pediatric Emergency Departments of a Level 1 Trauma Center in eastern North Carolina. Respondents reported household CBD use and selected from a list of conditions for which they take prescription medications. **Results**: Of 681 eligible respondents, 254 (37.3%) reported CBD use in their household (CBDUIH). Among those with CBDUIH, 69.7% reported concurrent use of 1 or more medications with a potential DDI risk. The most common categories of prescriptions were antidepressants (64.4%) and antihypertensives (41.8%), followed by agents for diabetes, hyperlipidemia, and immune disorders. **Conclusions**: The majority of CBD users in this population are concurrently taking medications with DDI potential, highlighting the need for patient and provider education, and improved labeling of CBD-based products to accurately reflect risks. Further study of clinically significant interactions is needed to determine which medications within these common categories have the most substantial risk of DDI.

## 1. Introduction

Cannabis is the most widely used illicit substance globally, with an estimated 181.8 million individuals aged 15–64 using it for nonmedical purposes in 2013 [1]. As legalization of cannabis has occurred in the United States, so too has its consumption and the consumption of its derivative products such as CBD [1]. In 2022, the National Survey on Drug Use and Health reported that an estimated 10.5% of the United States population reported using CBD in the past 30 days, with 40.7% of individuals reporting current use [2].

CBD is a non-psychoactive component of cannabis that lacks tetrahydrocannabinol (THC), the compound responsible for cannabis’s euphoric effects [3]. CBD has rapidly transitioned from a niche alternative therapy to a mainstream consumer product in the United States, particularly following the 2018 Farm Bill, which legalized the sale of hemp-derived products containing less than 0.3% THC freely, without federal control or oversight [4]. Now, CBD can be found in a wide variety of forms including oils, tinctures, edibles, and topical preparations, sold nationwide in pharmacies, gas stations, and via online retailers [4]. Its widespread availability, coupled with substantial marketing and consumer perception of CBD as “safe”, has fueled a sharp rise in use across most demographic groups [5,6].

Consumers may be under the impression that CBD products are under the same level of regulation as other food and drug products, but the US Food and Drug Administration has yet to implement substantial regulation over CBD products [7]. The marketing strategies used by cannabidiol companies often focus on various potential health benefits, and while CBD is well known to possess certain anti-inflammatory and antioxidant properties, evidence for its use in pain, anxiety and sleep is very limited [7]. Despite this limited evidence, CBD products are often marketed as safe and effective remedies for a myriad of ailments [7]. One analysis of 39 FDA-issued warning letters to companies that produced CBD products from 2015–2019 found that 97% of CBD marketing violations were due to its marketing as an unapproved new drug for over 125 health conditions, including cancer, diabetes and arthritis [7].

Importantly, CBD’s safety profile is often misunderstood by consumers. Its unregulated production and marketing can lead consumers to believe these products carry minimal risk, particularly in comparison to THC-containing products. However, since the production of these products is unregulated, many of them may pose unknown risks due to potential contamination with THC or discrepancies in doses and concentrations. CBD product mislabeling, ambiguous side effect notices, or unlabeled amounts of THC within the CBD products all have the capacity to pose health effects on consumers [7]. CBD has many interactions with prescription medications, primarily via its activity as a CYP enzyme inhibitor, most potently affecting CYP 3A4, 2C19, 2D6, and 2C9 [8]. By inhibiting CYP enzymes, CBD can increase the levels of other medications in the body, causing those medications to reach supratherapeutic, possibly toxic levels [5,9,10]. For medications with narrow therapeutic windows, like warfarin, which is metabolized by CYP 2C9, concordant CBD use may lead to adverse effects, like increased risk of bleeding (Table 1) [11].

The FDA defines drugs with a known interaction with CBD as a “sensitive substrate,” or a “moderate substrate” based on the level of CYP inhibitor and subsequent drug increase based on that inhibition. A sensitive substrate is when a drug taken with CBD has an increase in Area Under Concentration Time Curve (AUC) of >/= 5-fold. A “moderate sensitive substrate” demonstrates an increase in AUC of 2–5-fold increase [12].

Despite increasing research on cannabinoid pharmacokinetics and dynamics, clinical evidence characterizing the extent of CBD and prescription medication co-use remains limited. This knowledge gap limits the ability of healthcare providers to identify and appropriately counsel at-risk patients on potential DDIs. This study aims to address this gap by characterizing the patterns of concurrent CBD and prescription drug use across a broad cross-section of emergency department patients and their families. By identifying the therapeutic classes most frequently co-consumed, we aim to thereby highlight patient populations at greatest risk and provide a foundation for targeted education for patients and providers alike.

## 2. Methods

**Figure 1:**
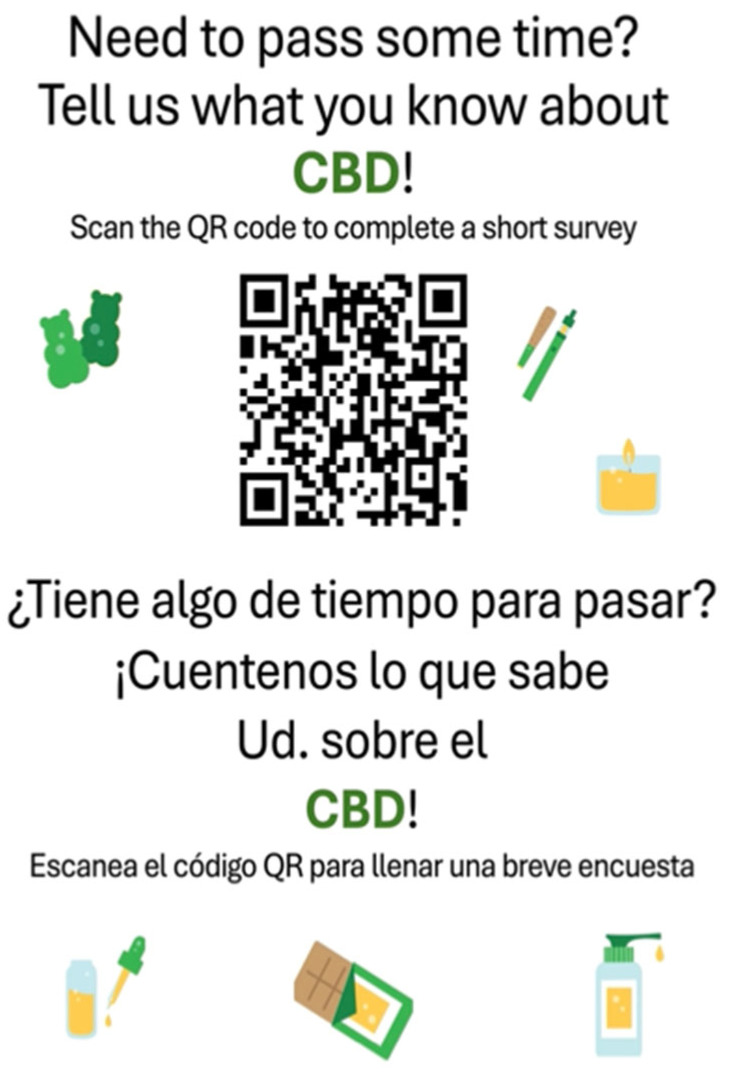
Distributed Flyer. Link accessed 1 November 2025; https://ecu.az1.qualtrics.com/jfe/form/SV_3UUUQnQfUw3ZCgC?Q_CHL=qr. The survey was available from 26 June 2024 through 15 May 2025.

A cross-sectional, observational study was performed at a rural, tertiary care academic medical center in the southeastern United States. The study setting included both the adult and pediatric Emergency Departments (EDs) of a Level 1 Trauma Center. Recruitment of participants occurred between August 2024 and May 2025. Flyers (Figure 1) containing a QR code linking to an online survey (hosted via QualtricsXM), were posted in publicly visible ED areas, including waiting rooms and patient bays, allowing voluntary participation from patients or accompanying family members. Recruitment was also carried out in-person, with several of the authors handing out flyers in these settings and encouraging participation. Device IP addresses were used by the Qualtrics software to prohibit duplicate responses. This study was approved by the University and Medical Center Institutional Review Board (UMCIRB# 24-000843) and deemed exempt.

The survey was available in English and Spanish and included multiple-choice and categorical items. Respondents were asked to provide basic demographic information (age only). Age was the only piece of personal information collected to limit participation hesitancy given CBD’s association with marijuana often stigmatized as a drug of abuse. They were asked to indicate whether anyone in their household uses CBD-containing products, and which types of prescription medications they or household members were currently taking. For accessibility reasons, medication information was collected using a predefined list of common condition categories (e.g., “depression/anxiety/mental illness”, “high blood pressure”, “high cholesterol”, “diabetes”, “immune condition”, “heart problem”, etc.) rather than by specific drug name or class. Medication classes were included in the list if one or more medications in that class (a) is known to have a potential DDI risk with CBD due to predominant metabolism by CYP3A4, 2C19, 2D6, or 2C9 according to the FDA CYP Enzyme Drug Table [12] and (b) is within the first 100 of the top 200 drugs by prescription number in the United States in 2023 according to the ClinCalc Drug Stats Database, which draws from the annual AHRQ Medical Expenditure Panel Survey [13]. Two authors independently verified the categorization to reduce bias. Responses from individuals under the age of 18 were excluded from data analysis. No personal identifying information was collected.

The primary exposure variable was self-reported household use of any CBD-containing product, regardless of source or formulation, which was treated as a binary variable. The primary outcome variable was concurrent use of a prescription medication with a known or potential DDI risk when combined with CBD, also treated as a binary variable.

Descriptive statistics were used to summarize respondent demographics and proportions of CBD use and concurrent prescription medication use. Results were reported as counts and percentages for categorical variables. Descriptive statistics were calculated using Qualtrics and Microsoft Excel. All responses indicating both CBD use and concurrent use of one of the listed categories of prescription medications were considered to represent a potential risk for drug–drug interaction.

## 3. Results

A total of 826 surveys were completed. 45 (5.4%) were excluded due to the respondent being <18 years of age, leaving 781 responses for analysis. Respondents ranged from age 18 to 85 with a median age of 34 (mean 37.5 ± 14.3). 95 respondents did not indicate whether CBD was used in their home, and were excluded from further analysis, leaving 681 respondents included in the final analysis.

**Figure 2:**
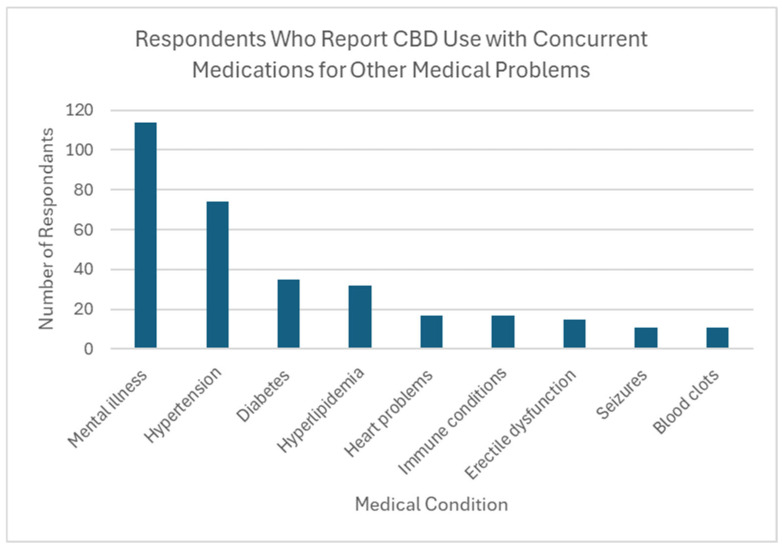
Respondents who report CBD use with concurrent medications for other medical problems.

Thirty-seven percent of respondents reported CBD use by someone in their household (*n* = 254). Over two thirds of these individuals (69.7%) reported household use of 1 or more types of prescription medication with a known interaction with CBD, creating potential DDIs in this population. The most common medications being taken were those for mental health conditions (*n* = 114, 64.4% of CBD users), followed by antihypertensives (*n* = 74, 41.8% of CBD users). Also reported was the use of medications to treat diabetes, high cholesterol, and immunologic conditions (Figure 2).

Of the 254 respondents reporting CBD use, most (*n* = 171, 67.3%) reported that their CBD products had not been recommended by a medical provider. Only 60 (23.6%) reported that their products had been medically recommended, while 23 (9.1%) elected not to answer this question (Table 2).

**Figure 3:**
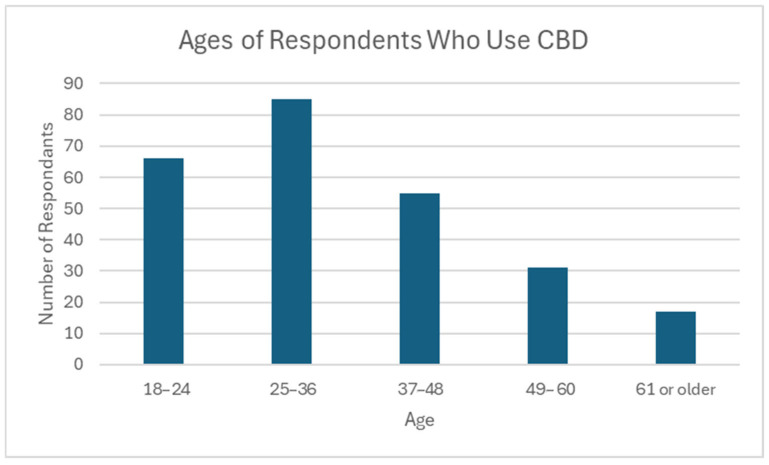
Ages of respondents who use CBD. *N* = 254; Minimum: 18; Maximum: 74; Mean: 35.9; Median: 33; Standard Deviation: 13.5.

Most CBD users in this survey were under the age of 49 (*n* = 206, 81.1%), with the oldest being 74 years of age (mean 35.9, median 33). The largest age group represented was 25–36 (*n* = 85, 33.5%). Only 6.7% of respondents who use CBD were over the age of 60 (*n* = 17) (Figure 3).

## 4. Discussion

Cannabidiol (CBD) is widely available over the counter for both recreational use and for presumed therapeutic purposes [1]. Emerging clinical evidence suggests that CBD possesses broad pharmacological potential, including anti-inflammatory, antioxidant, anticonvulsant, and anxiolytic properties [1,5]. There are multiple methods of use, including oral, transdermal, and inhalational formulations. However, the pharmacokinetic profile of CBD is complex, and its absorption and systemic effects are difficult to predict due to its poor water solubility, extensive hepatic metabolism, gastric instability, and erratic bioavailability [14]. Furthermore, the lack of consistent governmental regulation and oversight has led to poorly standardized product contents, contamination with delta-9 THC and other cannabinoids, and insufficient labeling regarding safe doses, contents, contraindications, and potential drug–drug interactions [14].

The intrinsic pharmacological activity of CBD can cause DDIs with commonly prescribed medications [5,10]. Its primary active metabolite, 7-hydroxy-CBD (7-OH-CBD), modulates cytochrome P450 (CYP450) enzymes, which play a central role in the metabolism and clearance of many pharmaceutical agents [9]. CBD and its metabolites interact with key CYP isoforms, particularly CYP3A4 and CYP2C19, which are responsible for its own biotransformation [9]. However, CBD also affects several other CYP enzymes and drug transporters involved in metabolite clearance, most potently CYP2C9 and CYP2D6. Inhibition of these four important enzymes can result in elevated plasma concentrations of dozens of medications who rely on these enzymes for their metabolism. Some of these medications include benzodiazepines and opioids, thereby increasing the risk of sedation and respiratory depression [5,10]. Similarly, concurrent use of CBD with antiepileptic drugs like valproate may exacerbate hepatotoxicity through enzyme inhibition and subsequent drug accumulation [5,10]. Table 2 describes the specific medications metabolized by the four CYP enzymes most potently inhibited by CBD (3A4, 2C19, 2D6, 2C9). These are the drugs that would be at greatest increased risk of potentially toxic level elevation with concurrent CBD use due to inhibition of their metabolism.

This study highlights a significant need for patient and provider education regarding potential DDIs between CBD and prescription medications. This study found that 69.7% of individuals reporting CBDUIH also take prescription medications with known potential risk for DDI. As the use of CBD products becomes increasingly widespread, the prevalence of potential drug interactions is likely to increase as well. Many individuals self-administer CBD, often to manage mental health conditions such as anxiety or depression [15,16]. This raises a particular concern for interactions between CBD and medications used to treat psychiatric disorders, especially given that mental health medication was the most commonly co-used medication class among those with CBDUIH.

Because of the potential for interactions with prescription medications, it is also important that health care providers ask patients about their use of CBD and educate them on the risks of combining CBD with certain prescription medications. The clinical significance of specific CBD-related interactions may guide how providers communicate these risks to their patients. Notably, the most common mechanism of interaction, CYP enzyme inhibition, can disrupt the metabolism of numerous drugs, increasing their systemic exposure and risk of toxicity. This is especially critical during the initiation of new prescription medications, or in patients taking medications with narrow therapeutic windows, such as warfarin or antiepileptic medications.

Unfortunately, there are few rigorous studies examining interactions between CBD and prescription medications in vivo; however, there have been several case reports and in vitro studies demonstrating elevation of plasma medication levels, including medications as common as escitalopram and warfarin [11,17]. One randomized-controlled trial (RCT) involving patients on anti-epileptic medications showed significant dose-dependent changes to serum levels of all medications studied in response to CBD administration; furthermore, the study showed elevation in liver transaminases in patients concomitantly taking valproate, possibly due to elevated medication levels [18]. Importantly, at least one RCT has shown transaminase elevation in healthy participants without concomitant medication use, implying that the hepatotoxicity of CBD may be magnified when co-administered with other medications like valproate that are known to be hepatotoxic [19]. The most rigorously studied category of medications with regard to DDIs with CBD is anti-epileptic medications; meta-analyses of trials in this patient population have shown increased plasma levels of common medications, such as clobazam and levetiracetam [9,20].

Our study suggests a substantial gap in patient and potentially provider understanding regarding CBD use and the potential for drug–drug interactions. Addressing this gap is essential for ensuring patient safety in the context of increasing CBD consumption. The current study suggests a substantial gap in patient and potentially provider understanding regarding CBD use and the potential for drug–drug interactions. Addressing this gap is essential for ensuring patient safety in the context of increasing CBD consumption. Additionally, more federal regulation is needed to standardized cannabidiol products and encourage companies to provide adequate labeling and warnings on CBD products to better inform consumers [14]. As the production of CBD products continues to increase and their incorporation into the alternative health movements becomes more large-scale, consumers need to have adequate safety measures in place to ensure that they understand the potential risk of using CBD products.

There are multiple potential imitations in this study. These results came from a geographic location in the rural southeastern United States. Larger studies across wider geographic areas are necessary to determine if the concurrent use of CBD and prescription medications varies based on geographic factors, socioeconomic factors, or if population usage differs in metropolitan or suburban areas. Additionally, our survey did not collect demographic information beyond the age of the respondent. This was an intentional attempt to increase the response rate by making respondents feel safe to participate by limiting personal information gathered. However, it will be important in the future to define if there is a specific demographic most at risk for DDIs so that educational efforts can be effectively targeted.

## 5. Conclusions

A significant gap in patient education regarding CBD–drug interactions is evident. Addressing this issue will require coordinated efforts from multiple stakeholders. Healthcare providers can play a critical role by proactively counseling patients on the potential for drug–drug interactions associated with both prescribed medications and over-the-counter CBD products. Additionally, efforts to ensure accurate labeling and clear communication of potential interactions with commonly prescribed drugs are needed. Standardized labeling and public health campaigns could help fill the current knowledge gap to promote consumer and patient safety.

## Figures and Tables

**Table 1:** Medications metabolized by CBD-inhibited enzymes.

CBD CYP Enzyme Inhibition	Drugs with Interaction
Sensitive Substrate CYP 3A4	Ticagrelor, Alfentanil, Avanafil, Budesonide, Buspirone, Conivaptan, Darifenacin, Darunavir, Dasatinib, Dronedarone, Eletriptan, Eplerenone, Everolimus, Felodipine, Ibrutinib, Indinavir, Isavuconazole, Ivabradine, Leemborexant, Lomitapide, Lovastatin, Lurasidone, Maraviroc, Midazolam, Mobocertinib, Naloxegol, Nisoldipine, Saquinavir, Sildenafil, Simvastatin, Sirolimus, Tacrolimus, Tipranavir, Tolvaptan, Triazolam, Vardenafil, Venetoclax, Quetiapine
Moderate Substrate CYP 3A4	Atorvastatin, Aprepitant, Colchicine, Eliglustat, Alprazolam, Pimozide, Rilpivirine, Rivaroxaban, Tadalafil, Selpercatinib, Tazemetostat
Sensitive Substrate CYP 2C19	Omeprazole, s-Mephenytoin
Moderate Substrate CYP 2C19	Diazepam, Lansoprazole, Rabeprazole, Voriconazole
Sensitive Substrate CYP 2D6	Atomoxetine, Desipramine, Dextromethorphan, Eliglustat, Nebivolol, Perphenazine, R-venlafaxine, Tolterodine
Moderate Substrate CYP 2D6	Imipramine, Metoprolol, Nortriptyline, Propafenone, Propranolol, S-venlafaxine, Tramadol, Trimipramine
Sensitive Substrate CYP 2C9	Celecoxib
Moderate Substrate CYP 2C9	Glimepiride, Phenytoin, Tolbutamide, Warfarin

**Table 2:** Number of respondents who have been recommended CBD products by a medical professional.

Recommended CBD by Medical Professional?	Count (%)
Yes	60 (23.9)
No	171 (67.3)
Did Not Answer	23 (9.1)

## Data Availability

The raw data supporting the conclusions of this article will be made available by the authors on request.
